# BRD9 binds cell type-specific chromatin regions regulating leukemic cell survival via STAT5 inhibition

**DOI:** 10.1038/s41419-019-1570-9

**Published:** 2019-04-18

**Authors:** Nunzio Del Gaudio, Antonella Di Costanzo, Ning Qing Liu, Lidio Conte, Antimo Migliaccio, Michiel Vermeulen, Joost H. A. Martens, Hendrik G. Stunnenberg, Angela Nebbioso, Lucia Altucci

**Affiliations:** 1 Department of Precision Medicine, University of Campania “Luigi Vanvitelli”, Vico L. De Crecchio 7, 80138 Napoli, Italy; 2 Division Gene Regulation, Netherlands Cancer Institute, Plesmanlaan 121, 1066 CX Amsterdam, The Netherlands; 3 Department of Molecular Biology, Faculty of Science, Radboud Institute for Molecular Life Sciences, Oncode Institute, Radboud University, 6525 GA Nijmegen, The Netherlands

**Keywords:** Cancer therapy, Leukaemia, Epigenetics

## Abstract

Leukemia is characterized by genetic and epigenetic mutations resulting in selection of cancer cells, which are unable to differentiate. Although genetic alterations are difficult to target, the epigenome is intrinsically dynamic and readily offers new therapeutic strategies. Thus, identifying cancer-specific context-dependent targets and unraveling their biological function may open up new therapeutic perspectives. Here we identify bromodomain-containing protein 9 (BRD9) as a critical target required in acute myeloid leukemia (AML). We show that BRD9 is overexpressed in AML cells including ex vivo primary blasts compared with CD34^+^ cells. By targeting BRD9 expression in AML, we observed an alteration in proliferation and survival, ultimately resulting in the induction of apoptosis. Intriguingly, genome-wide profiling revealed that BRD9 binds enhancer regions in a cell type-specific manner, regulating cell type-related processes. We unveil a novel BRD9-sustained STAT5 pathway activation via regulation of SOCS3 expression levels. Our findings identify a previously undescribed BRD9-STAT5 axis as critical for leukemia maintenance, suggesting BRD9 as a potential therapeutic target.

## Introduction

Leukemia is a hematological malignancy characterized by neoplastic clones that are unable to differentiate^1^. Although recurring protein-coding mutations and chromosomal aberrations are essential to leukemic pathogenesis, epigenetic mutations critically contribute to its development and/or maintenance^2^. DNA methylation and histone posttranslational modification machinery together with proteins specialized for the interpretation of histone modification (readers) all contribute to leukemogenesis. Readers are defined as chromatin regulators possessing specific domains that recognize and bind covalent modifications of nucleosomes^3^. Bromodomain-containing proteins (BRDs) specifically bind acetylated lysines via an evolutionarily conserved protein interaction module. BRDs are principally involved in gene transcription regulation, cell cycle control, cell growth, DNA damage response, inflammation, and development^4,5^.

Several BRDs, functioning as either oncogenes or tumor suppressors (TSs), have been implicated in cancer development and maintenance, making them attractive pharmacological targets for future anticancer strategies^6^. For example, overexpression of the histone readers ATAD2 and TRIM24 was associated with poor overall survival in breast cancer^7^, whereas BRDT protein was frequently found overexpressed in non-small cell lung cancer and other low survival cancers^8^. In addition, two different short hairpin RNA (shRNA) screening-based studies identified BRD4 and SMARCA4 as targets for acute myeloid leukemia (AML) development and maintenance, sustaining the transcription of *MYC* and *HOXA9/MEIS*, respectively, and leading to abnormal differentiation, proliferation, and survival^9,10^.

Besides SMARCA4, other SWI/SNF subunit proteins with a BRD module were shown to be involved in cancer, including BRD7^11^. Several studies described BRD7 as a TS, whose expression is downregulated in multiple cancer types including nasopharyngeal, endometrial, and hepatocellular carcinomas^12^, as well as ovarian and colorectal cancers^13^. BRD7 is also reported to interact with BRCA-1 and p53, contributing to suppression of breast cancer cell survival mechanisms^14^. Although the role of BRD7 in cancer has been extensively studied, the biological function and involvement in human malignancies of its close homolog BRD9 has not yet been elucidated. Here we identify BRD9 as critically required in AML. We reveal that BRD9 is overexpressed in AML cells, including ex vivo primary blasts, compared with CD34^+^. In support of the leukemogenic role of BRD9, shBRD9-expressing AML cells displayed a strong impairment of proliferation and survival, accompanied by induction of apoptosis. Interestingly, BRD9 preferably binds enhancer regions in a cell type-specific manner, regulating cell type-related processes. Our study of BRD9 epigenome regulation in AML supports the hypothesis that the effects of BRD9 suppression are due to its role in sustaining STAT5 pathway activation via SOCS3 expression level regulation. Our data provide new insights into the function of BRD9 in cancer and suggest BRD9 as a novel potential therapeutic target in leukemia.

## Results

### BRD9 is overexpressed in cancer

**Fig. 1:**
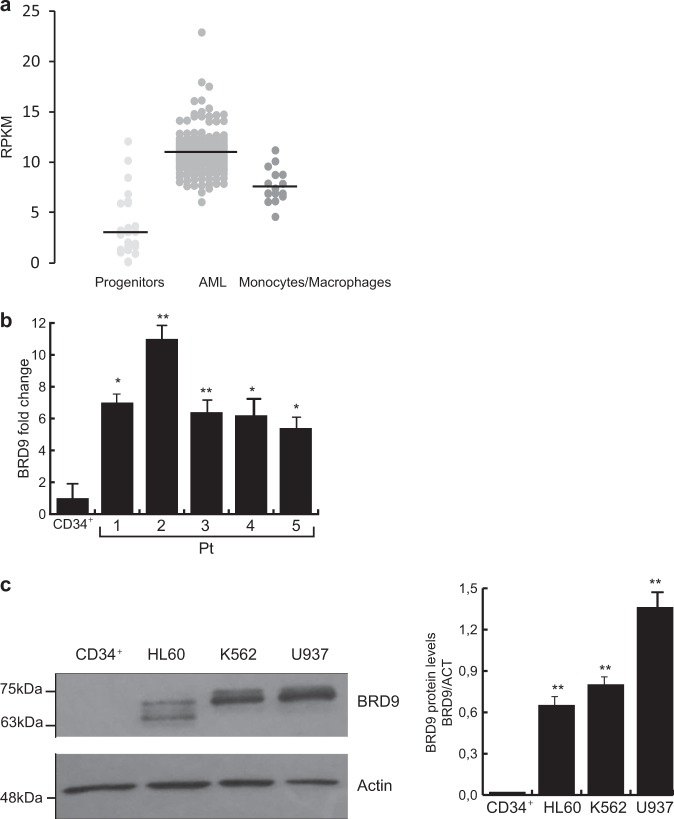
BRD9 is overexpressed in cancer. **a** Dot plot bioinformatics analysis showing BRD9 expression in publicly available RNA-seq data from 200 primary AML samples, 19 samples of human hematopoietic progenitors, and 16 samples of differentiated human monocyte and macrophage cells. β-Actin was used as housekeeping gene (*P* < 0.01 Welch’s *t*-test). **b** RT-qPCR analysis of BRD9 expression in primary AML samples (pt) compared with CD34^+^ cells; error bars indicate SD of three biological replicates (*P < 0.05, **P ≤ 0.01). **c** WB analysis of BRD9 in the indicated leukemic cell lines compared with the CD34^+^. Actin was used as loading control. Error bars indicate SD of three biological replicates (*P < 0.05, **P ≤ 0.01)

To investigate the involvement of BRD9 in human leukemia, we first analyzed BRD9 expression levels in 200 clinically annotated AML samples (The Cancer Genome Atlas (TGCA); Ley et al.^15^), 19 non-transformed human bone marrow progenitor samples (Hematopoietic Stem Cells (HSCs), MultiPotent Progenitors (MPPs), Common Myeloid Progenitors (CMPs), Common Lymphoid Progenitors (CLPs), Granulocyte-Monocyte Progenitors (GMPs), Megakaryocyte-Erythrocyte Progenitors (MEPs)), and 16 differentiated human hematopoietic cell samples (monocytes and macrophages) (Blueprint data set). The results were shown as dot plot representing BRD9 expression value in samples belonging to the specific cell-type subgroup. AML samples exhibited increased BRD9 expression compared with progenitors and differentiated cells, whereas no difference was observed between AML samples belonging to different cell subgroup based on World Health Organization leukemic classification (Fig. 1a, Supplementary Fig. 1a).

To corroborate BRD9 overexpression in AML, we examined BRD9 expression levels in five ex vivo primary human AML blasts compared with CD34^+^. BRD9 expression was significantly higher in all leukemic blasts than in CD34^+^ cells (Fig. 1b).

To further confirm this finding, we evaluated BRD9 protein levels in a panel of human hematological and solid cancer cell lines compared with CD34^+^ and non-transformed cells (primary endometrial cells), respectively. BRD9 protein levels were dramatically increased in all cancer/tumorigenic cells analyzed. In contrast, very low levels of BRD9 protein were detected in normal and CD34^+^ progenitors (Fig. 1c, Supplementary Fig. 1b, c).

Taken together, these data indicate that BRD9 is overexpressed in both leukemia and solid cancer cells.

### AML cells are sensitive to BRD9 depletion

**Fig. 2:**
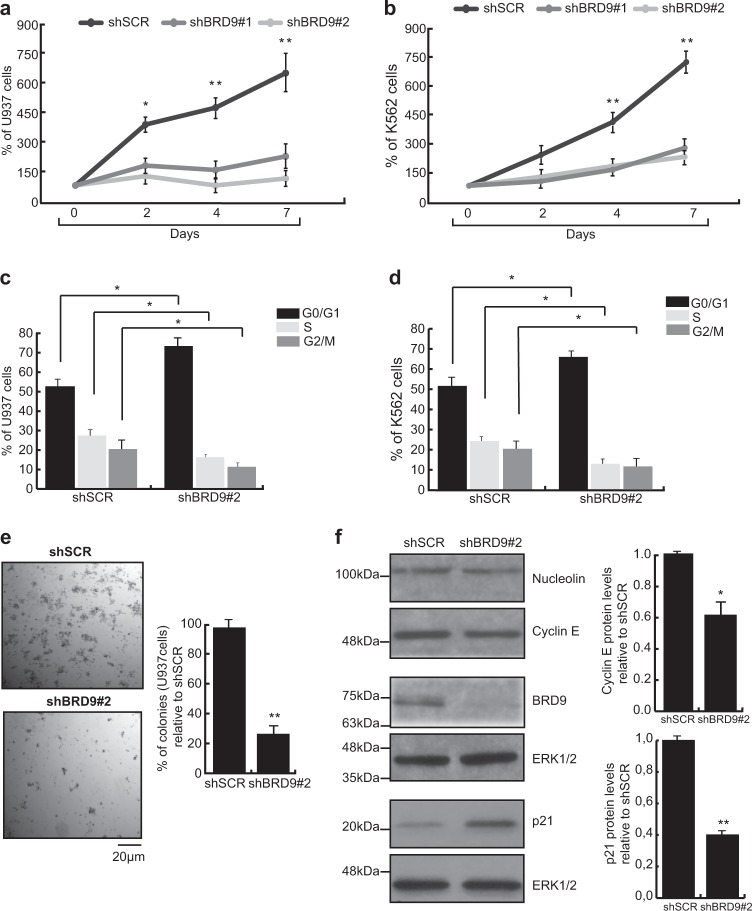
BRD9 depletion affects leukemic cell survival and proliferation. **a**, **b** Competitive proliferation by Trypan blue exclusion assay of shBRD9- and shSCR-transduced U937 (**a**) and K562 cells; error bars indicate SD of three biological replicates. **c**, **d** FACS analysis of U937 (**c**) and K562 (**d**) cells following shBRD9 or shSCR transduction; analysis was performed after 5 days upon puromycin selection; error bars indicate SD of three biological replicates. **e** Colony-formation capacity assay of shBRD9- and shSCR-transduced U937 cells; results are shown as % of colonies generated by BRD9 KD cells relative to shSCR-transduced cells; error bars indicate SD of three biological replicates (*P < 0.05, **P ≤ 0.01). **f** WB showing expression levels of the indicated proteins after 3 days upon puromycin selection. ERK1/2 was used as loading control. Error bars indicate SD of three biological replicates (*P < 0.05, **P ≤ 0.01)

To investigate the potential involvement of BRD9 in leukemia, we assayed the biological effects of shRNA-mediated BRD9 knockdown (KD) on leukemic cell growth. U937, K562 HL-60, KASUMI, and NB4 cells were transduced with a lentivirus expressing shBRD9#1, shBRD9#2, or non-targeting shRNA (shSCR) (Supplementary Fig. 1d, e, f, g and h). After puromycin selection, proliferation of BRD9 KD cells was significantly reduced compared to scrambled control (Fig. 2a, b, Supplementary Fig. 2a, d). In addition, shBRD9-expressing cells were arrested in G0/G1 phase of cell cycle and showed a reduction in S phase compared to shSCR-transduced cells (Fig. 2c, d). Similarly, the colony-forming capability of U937 cells was also significantly reduced following BRD9 depletion compared with control (Fig. 2e). In agreement with the phenotypic results, shBRD9-expressing U937 cells revealed overexpression of p21 and downregulation of cyclin E compared with shSCR (Fig. 2f).

Taken together, these findings indicate that BRD9 is required for clonogenicity and proliferation of myeloid leukemic cell lines, suggesting a possible induction of shBRD9-mediated cell death.

### BRD9 depletion induces apoptosis of cell lines and ex vivo primary AML blasts

**Fig. 3:**
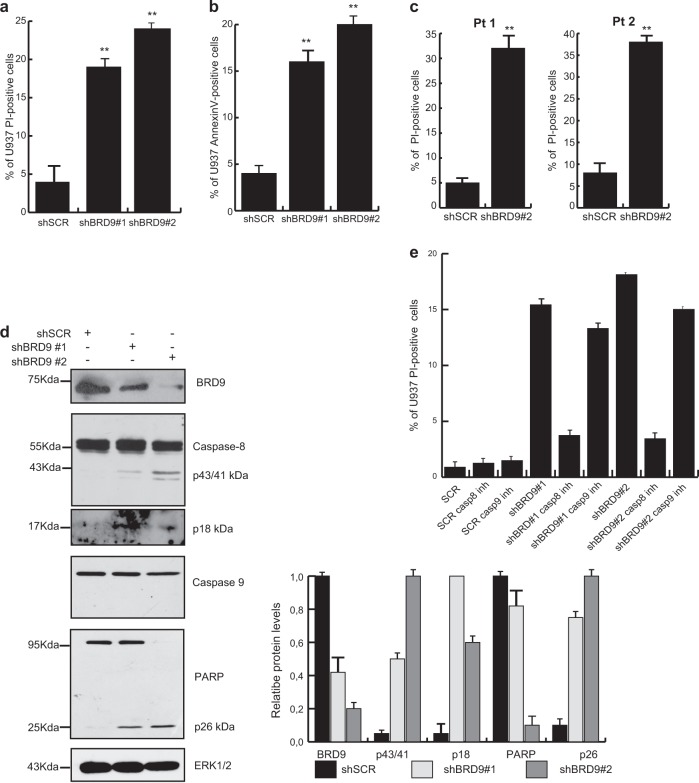
BRD9 depletion induces apoptosis of leukemic cells. **a**, **c** Percentage of PI-positive cells following BRD9 depletion in (**a**) U937 cells and (**b**) two ex vivo leukemic samples (Pt 1 and Pt 2); error bars indicate SD of three (**a**) and two (**c**) biological replicates (*P < 0.05, **P ≤ 0.01). **b** Percentage of AnnexinV-positive U937 cells upon BRD9 KD; analysis was performed after 5 days upon puromycin selection; error bars indicate SD of three biological replicates (*P < 0.05, **P ≤ 0.01). **d** WB analysis showing levels of the indicated proteins in shBRD9- or shSCR-transduced U937 cells after 3 days upon puromycin selection. ERK1/2 was used as loading control. Immunoblottings were performed sequentially on the same membrane. Densitometric analysis is shown, error bars indicate SD of three biological replicates (*P < 0.05, **P ≤ 0.01). **e** PI analysis of shSCR- shBRD9#1- and shBRD9#2-transduced U937 treated with 100 μM of Caspase9 or Caspase8 inhibitors

To explore BRD9 KD-induced cell death, the fraction of propidium iodide (PI)-positive cells was measured. shBRD9-expressing U937, K562, HL-60, KASUMI, and NB4 cells displayed increased cell death percentages compared with shSCR (Fig. 3a and Supplementary Fig. 2b, c, e and f). We also investigated the sensitivity of ex vivo myeloid leukemic blasts to BRD9 KD. Two ex vivo primary AML samples were collected and transduced with a lentivirus expressing shBRD9 or shSCR. PI analysis was performed 48 h after transduction. shBRD9-expressing blasts showed a higher percentage of PI-positive cells than shSCR. (Fig. 3c). Next, to address apoptosis onset as a mechanism of observed cell death induction, protein levels of key apoptotic genes were analyzed. U937 cells expressing shBRD9 showed a strong induction of caspase8 via p43/41 kDa and p18 kDa fragments, and Poly (ADP-ribose) polymerase (PARP) via p27 kDa fragment. In contrast, neither pro-caspase8 nor PARP cleavage were observed in shSCR-transduced cells (Fig. 3d). In addition, we investigate the impact of caspase inhibition on BRD9 KD cells. By PI analysis of shBRD9-tranduced cells treated with Caspase8 and Caspase9 inhibitors, respectively, we observed that Caspase8 inhibition was able to rescue the shBRD9-induced cell death. Conversely, inhibition of Caspase9 failed to rescue the phenotype. Results confirmed that the BRD9 KD-induced apoptosis is Caspase8 dependent (Fig. 3e). Apoptosis onset following BRD9 depletion was also confirmed by Annexin-V staining assay in both U937 and KASUMI cells (Fig. 3b, Supplementary Fig. 2g, 3a, b).

Taken together, these findings indicate that BRD9 depletion induces apoptosis via Caspase8 activation in both in vitro and ex vivo primary AML cells.

### BRD9 is part of the SWI/SNF complex and targets putative enhancer regions in a cell type-specific manner

**Fig. 4:**
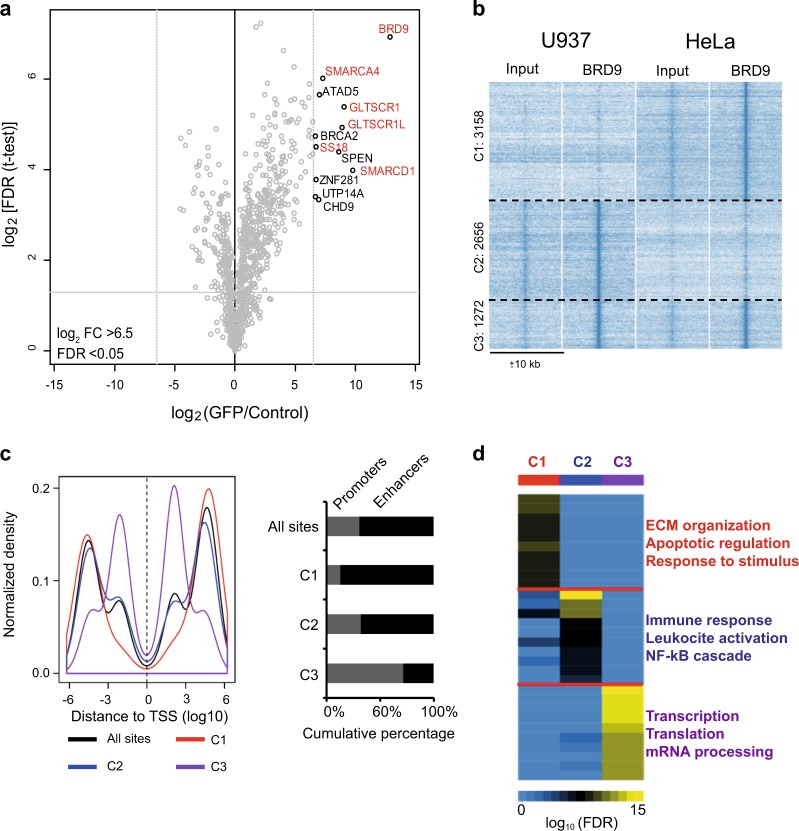
Genome-wide binding of BRD9 is highly cell type specific. **a** Volcano plot from label-free GFP pulldown of GFP-BRD9 HeLa cell nuclear extracts. Bait and its interactors are shown in the upper right corner (SWI/SNF members including BRD9 are shown in red). Statistically, enriched proteins in GFP-BRD9 pulldown were identified by a permutation-based FDR-corrected *t*-test. Label-free quantification intensity of GFP pull-down relative to control (fold change, *x*-axis) is plotted against the log2-transformed *p*-value of *t*-test (*y*-axis). BRD9 interacts with proteins previously reported as core member of the BAF-SWI/SNF complex. **b** K-means clustering analysis of BRD9 ChIP-seq data showing three distinct clusters of BRD9-binding sites with different specificity for U937 and HeLa cells. **c** Distance of the clusterized BRD9-binding sites to TSS (promoters: ≤1 kb from TSS, putative enhancers: >1 kb from TSS). **d** GO analysis of BRD9-binding sites using Genome regions enrichment of annotations tool (GREAT). The most significant GO terms for each of the three clusters are shown

To corroborate and strengthen the needed involvement of BRD9 in AML, we addressed its molecular role in cancer by mass spectrometry (MS)-based quantitative interaction proteomics. We generated a transgenic HeLa stable cell line overexpressing N-terminal green fluorescent protein (GFP)-tagged BRD9 protein, downstream a doxycycline-inducible promoter (Supplementary Fig. 3c). After 24 h of doxycycline induction, GFP pulldown of nuclear HeLa extracts was performed and precipitated proteins were analyzed by liquid chromatography (LC)-MS/MS. The results confirmed the interaction of BRD9 with proteins belonging to the SWI/SNF complex—SMARCA4, SS18, SMARCD1, and the recently identified SWI/SNF interactors GLTSCR1 and GLTSCR1L^16^ classifying BRD9 as a core member of the complex (Fig. 4a). In addition, we validated LC-MS/MS data by GFP pull-down assay following western blotting (WB) analysis of U937 cells expressing GFP-BRD9. Results confirmed the interaction of BRD9 with SMARCA4 (Supplementary Fig. 3d). Given that reader subunits belonging to SWI/SNF are expected to mediate correctly positioning of the complex on certain genomic regulatory regions such as enhancers^17^, we reasoned that BRD9 may also show a cell type-specific genomic binding pattern. Thus, to identify leukemic-specific BRD9-binding sites, we determined genome-wide BRD9 binding using chromatin immunoprecipitation-sequencing (ChIP-seq) in the U937 cell line and the non-hematopoietic HeLa cell line. The majority of BRD9-binding sites were cell type specific: U937 specific (Cluster C2, *n* = 2656) or HeLa specific (Cluster C1, *n* = 3158), respectively, whereas a smaller fraction of binding sites were detected in both cell lines (Cluster C3, *n* = 1272) (Fig. 4b). The cell type-specific BRD9-binding cluster C1 in HeLa and, to a lesser extent, C2 in U937 preferably located at putative enhancers (>1 kb of transcription start sites [TSS]), whereas the common binding cluster C3 showed a clear enrichment within promoters (≤1 kb of TSS) (Fig. 4c). We further validate ChIP-seq results by an independent ChIP-quantitative PCR (qPCR) experiment analyzing specific BRD9 targets (Supplementary Fig. 5d).

To reveal the potential function of BRD9-binding sites, we performed Gene Ontology (GO) analysis on the three clusters. GO terms associated with general cellular processes such as transcription and translation regulation were enriched in the common C3-binding cluster. In contrast, we found that C1 biological processes were associated with extracellular matrix organization and C2 GO terms were strongly linked to immune response, which are related to epithelial and hematopoietic cells, respectively. These data suggest that BRD9 binding at putative enhancers regulates cell type-specific processes, whereas at promoters it regulates common cellular processes (Fig. 4d).

Taken together, these findings highlight that BRD9 belongs to the SWI/SNF complex and binds certain enhancer elements in a cell type-specific manner.

### BRD9-binding sites are mainly associated with active chromatin regions

**Fig. 5:**
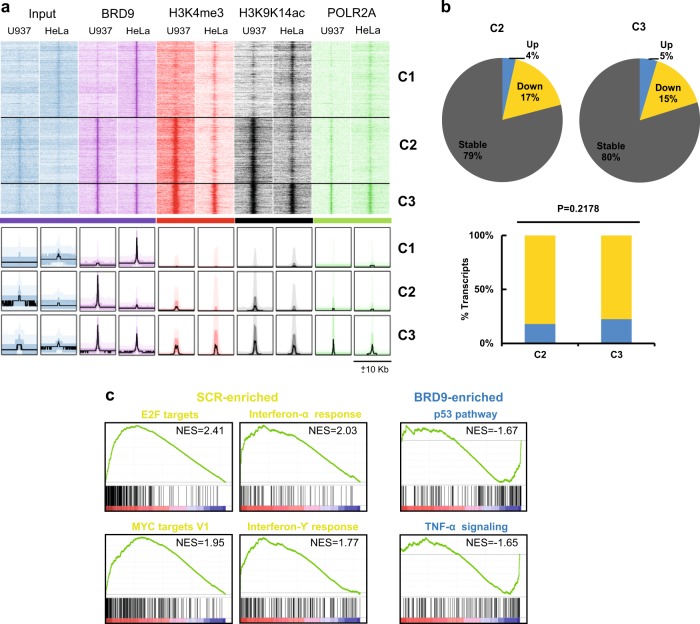
Transcriptomic analysis identifies anti-proliferative and apoptotic pathways induced by BRD9 depletion. **a** Genome-wide heatmap analysis indicating the co-occurrence of BRD9-binding sites with active chromatin regions. **b** Pie chart showing dominant gene silencing observed upon BRD9 KD for genes associated with C2 and C3, suggesting BRD9 as a transcription activator. However, there is no significant difference between numbers of activated and inactivated genes in C2 and C3 clusters. **c** GSEA analysis showing the top differential hallmark gene sets (nominal *P* < 0.05) associated with SCR and shBRD9 cells (NES = normalized enrichment score)

We next examined the chromatin environment at BRD9-binding regions. Using an integrative data analysis of BRD9, active histone markers (H3K4me3, recently shown to occur also at active enhancer level^18^ and H3K9K14ac), and POLR2A occupancy, we found that BRD9 co-occurs at active chromatin sites in both U937 and HeLa cells (Fig. 5a). Furthermore, we observed transcription levels of genes associated with BRD9-binding sites in shBRD9-transduced U937 cells. BRD9 KD resulted in dominant gene silencing in both the U937-specific C2 cluster (4% upregulation compared with 17% downregulation) and the universal C3 cluster (5% upregulation compared with 15% downregulation) (Fig. 5b, Supplementary Fig. 4a, b). However, a small percentage of in cis BRD9-regulated genes was also found actively transcribed, including sets of genes positively regulating the apoptotic pathway (Fig. 5b, Supplementary Fig. 4a, b).

Taken together, these data highlight that BRD9 is mainly associated with active chromatin regions, functioning primarily as a transcriptional activator.

### BRD9 depletion affects key proliferation and immune response pathways in AMLs

**Fig. 6:**
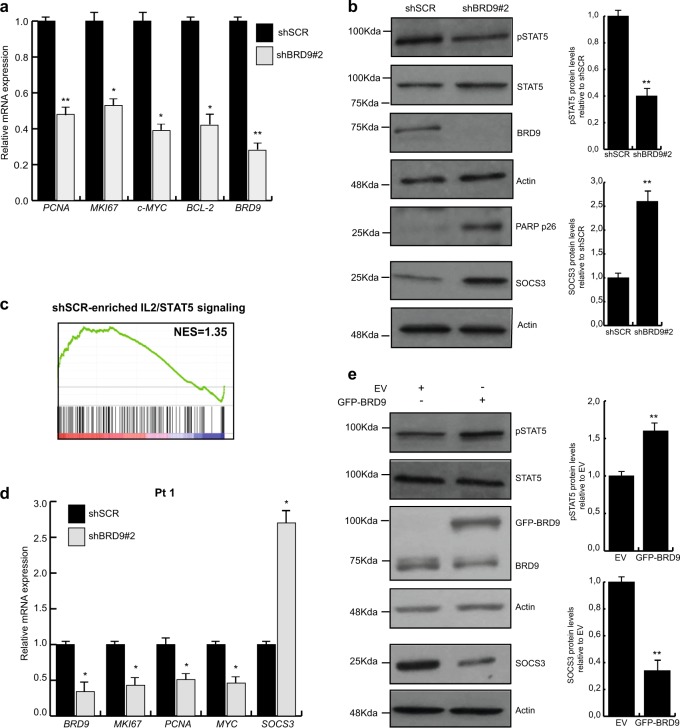
BRD9 depletion affects STAT5 pathway activation. **a** RT-qPCR of indicated genes in shBRD9-transduced U937 cells. Error bars indicate SD of three biological replicates (*P < 0.05, **P ≤ 0.01). **b** WB analysis of indicated proteins upon BRD9 depletion in U937 cells after 3 days upon puromycin selection. Immunoblottings were performed sequentially on the same membrane. Error bars indicate SD of three biological replicates (*P < 0.05, **P ≤ 0.01). **c** GSEA showing STAT5 hallmark gene set (*P* < 0.05) associated with shSCR- and shBRD9-transduced U937 cells. **d** RT-qPCR showing relative expression levels of indicated genes. Experiment was performed 48 h following shBRD9 transduction of ex vivo leukemic cells (Pt 1). Error bars indicate SD of two biological replicates (*P < 0.05, **P ≤ 0.01). **e** WB analysis of indicated proteins performed 48 h following BRD9 overexpression in U937 cells. Immunoblottings were performed sequentially on the same membrane. Error bars indicate SD of three biological replicates (*P < 0.05, **P ≤ 0.01)

To identify BRD9-associated pathways, we performed RNA sequencing (RNA-seq) on scramble- and shBRD9-transduced U937 cells. A total of 2113 genes were deregulated with ≥2-fold difference in expression, 412 of which were upregulated and 1701 downregulated in shBRD9 compared with shSCR-transduced cells. Using gene-set enrichment analysis (GSEA) we identified the top differential expressed gene sets up- and downregulated in shBRD9- compared with shSCR-transduced cells. shBRD9 caused the downregulation of gene sets related to proliferation (such as E2F and MYC targets) and immune response (such as INFα and γ-targets). In contrast, the gene sets upregulated in shBRD9 cells involved apoptotic pathways (such as p53 and tumor necrosis factor-α targets) (Fig. 5c and Supplementary Fig. 4a and c). We validated RNA-seq results of the key deregulated genes using reverse transcriptase-qPCR (Fig. 6a and Supplementary Fig. 4b).

Altogether, these results show that BRD9 KD affects key proliferative pathways in AML.

### BRD9 promotes AML cell survival primarily via STAT5 activation

Among the in cis BRD9-target genes deregulated following BRD9 KD, *SOCS3* was identified as one of the top upregulated (fold change > 4.5). As a consequence, we hypothesized that SOCS3 might be a solid candidate for mediating the shBRD9-induced phenotype. SOCS3 negatively regulates Janus kinase family members, which inhibits the activation of STAT proteins, including STAT5. Activation of STAT5 has been implicated in the stimulation of AML proliferation and survival, as well as in inflammation^19–21^. We validated SOCS3 upregulation upon BRD9 depletion at protein (Fig. 6b) and mRNA level (Supplementary Fig. 4b); we also confirmed that BRD9 localizes at SOCS3 regulative regions (Supplementary Fig. 5c and d). We corroborated the impairment of STAT5 activation by detecting low levels of phosphorylated STAT5 (pSTAT5) (Fig. 6b). Reduced pSTAT5 levels resulted in the downregulation of key proliferative (*PCNA*, *MYC*, *MIK67*) as well as STAT5-regulated (*MYC*, *BCL2*) genes (Fig. 6a)^22^, and in the induction of apoptosis assayed by PARP-cleavage fragment p26 (Fig. 6b). In line with these observations, downregulation of the gene set related to activation of STAT5 pathway was highlighted by GSEA (Fig. 6c). Upregulation of *SOCS3* and downregulation of *PCNA*, *MYC*, *MIK67*, and *BCL2* genes were also found in both ex vivo shBRD9-transduced leukemic samples (Fig. 6d and Supplementary Fig. 4a).

To further investigate the involvement of BRD9 in regulating the STAT5 pathway, we overexpressed GFP-BRD9 in U937 and K562 cell lines. As expected, lower SOCS3 and higher pSTAT5 protein levels were observed in BRD9-enriched cells than in control, indicating the BRD9-mediated activation of STAT5 pathway supporting AML tumorigenesis (Fig. 6e and Supplementary Fig. 5b).

Taken together, these results show that BRD9 is a key regulator for STAT5 activation in leukemia via regulation of SOCS3 expression.

## Discussion

In the present study we identify BRD9 as a key regulator of AML tumorigenesis and offer new insights into the role of BRD9 in hematological malignancies. We showed that the expression of BRD9 is higher in both primary and leukemic cell lines than in CD34^+^ cells. By targeting BRD9, we provided evidence that BRD9 regulates AML cancer cell proliferation and tumorigenicity, indicating its proto-oncogenic role in transformed blood cells. In support of these findings, we identified impairment of cell cycle progression and induction of apoptosis pathways via caspase8 activation as the most prominent phenotypic effects upon BRD9 KD. We also analyzed induction of differentiation following BRD9 depletion, but, in contrast with a previous study^23^, we did not observe leukemia cell differentiation.

We identified SWI–SNF complex members as the strongest interactors of BRD9, indicating its involvement in chromatin remodeling and transcriptional regulation. Intriguingly, by analyzing BRD9 chromatin-wide binding sites we found that BRD9 binding mainly occurs at the enhancer level in a cell type-specific manner, regulating cell type-related processes. It is interesting to speculate that BRD9-related processes might be responsible for cell identity. Specifically, BRD9 chromatin binding in AML mainly regulates immune response-related genes. Conversely, at promoter level, BRD9 primarily co-occurs at the same genomic sites in different cell types, regulating common cellular processes such as transcription. Our findings are in agreement with a recent publication identifying the SWI/SNF subunit member SMARCB1 as required to target the SWI/SNF to specific enhancer regions and provide new insights into BRD proteins to a cancer-related SWI/SNF function. However, the role of BRD9 and its cell-context dependency in other cancers and diseases still needs to be addressed.

To explore BRD9 upregulation in leukemia, we analyzed epigenetic marks in BRD9 regulatory regions of AML patient’s cohort compared with normal progenitors and differentiated cells; unfortunately, we did not highlight significative differences between them. Thus, BRD9 upregulation in leukemia could be due to a genetic alteration or overexpression of “positive BRD9 regulators”. The combination of proteomic experiments in different leukemic cell lines and BRD9 motifs analysis may help in addressing these remaining open questions.

Depletion of BRD9 alters the transcription program of leukemic cells, inducing enrichment of cell death pathways and downregulation of genes involved in cell survival. Among the small percentage of overexpressed in cis BRD9-regulated genes, we identified SOCS3 as a prominent target responsible for the observed BRD9-depleted phenotype. Furthermore, we showed for the first time that by negatively regulating SOCS3 expression, BRD9 in turn influences activation of the tumor-driver STAT5 pathway, affecting leukemic cell proliferation and survival. Our findings are also supported by a recent report describing that inhibition of STAT5 is associated with apoptosis induction via Caspase8 activation^24^. Accordingly, inhibition of BRD9 in leukemia reduces STAT5 activation and induces apoptosis via Caspase8 and not Caspase9 cleavage. The STAT5 pathway and its involvement in cancer initiation and progression has been extensively studied^25^. Although major efforts are being directed toward the development of inhibitors targeting STAT molecules, particularly STAT5, candidate compounds have not yet shown sufficient promise to advance to clinical trials^26^. Consequently, inhibiting BRD9 may represent not only a possible approach to targeting leukemia but also an alternative strategy to target pSTAT5-driven tumors. Moreover, as it was shown that healthy cells are able to survive at low levels of STAT5 activation^27^, targeting STAT5 via BRD9 inhibition may also represent a very promising approach for developing anticancer compounds displaying low toxicity.

Recently, BRD9 inhibitors (BRD9i) such as LP99 and I-BRD9 were synthetized^28,29^. These molecules display selective affinity for BRD9, inhibiting its binding to chromatin. We treated leukemic cells with LP99, but we did not observe a significant alteration of cell proliferation.

Although other BRD9i showing a higher affinity to BRD9 were very recently synthetized and should thus be tested in future investigations^12^, an intriguing explanation for LP99 failure in our settings might be attributed to the large diversity of protein interaction domains or to a combination of interaction domains that are able to anchor the SWI/SNF complex to specific sets of chromatin sites independently of BRD9. This hypothesis is also supported by recent evidence showing that PFI-3, a selective SMARCA4/2 BRDi, fails to phenocopy the observed SMARCA2-depleted phenotype in SMARCA4-deficient lung cancer cells.

Using BRD9i alone or in combination with other SWI/SNF and reader inhibitors may nevertheless represent a potential new approach for future anticancer therapy^30,31^.

Collectively, our findings identify BRD9 as a driver sustaining proliferation and survival of leukemic cells, and point to its potential as a therapeutic target in leukemia.

## Materials and methods

### Cell culture

K562, U937, HL-60, KASUMI, NB4, OCI-AML2, MOLM-14, THP, and KG-1 cells (DMSZ, Germany) were cultured in RPMI 1640 (Euroclone, Italy) supplemented with 10% heat-inactivated fetal bovine serum (FBS) (Sigma-Aldrich, Italy), 2 mM l-glutamine (Euroclone), and antibiotics (100 U/mL penicillin, 100 μg/mL streptomycin, and 250 ng/mL amphotericin-B). HEK293FT, U87MG, HeLa, HCT116, and HCT116 p53^−/−^ cells were grown in Dulbecco’s modified Eagle’s medium (DMEM) (Euroclone) supplemented with 10% FBS, 100 U/mL penicillin/streptomycin (Euroclone), and 6 mM (HEK293FT) or 2 mM glutamine (Euroclone). Transgenic HeLa cells overexpressing GFP-BRD9 were cultured in DMEM supplemented with heat-inactivating FBS, 2 mM l-glutamine, 1% penicillin/streptomycin, 50 μg/mL hygromycin (Thermo Fisher Scientific, Italy), and 2 μg/mL blasticidin S (Sigma-Aldrich).

### Primary cell culture

Leukemic blasts cells were recovered from the peripheral blood or bone marrow of leukemic patients and purified by Ficoll density gradient separation (Sigma-Aldrich). Cells were cultured in RPMI 1640 (Euroclone) supplemented with 20% heat-inactivated FBS (Sigma-Aldrich), 1% glutamine (Euroclone), 1% penicillin/streptomycin (Euroclone), and 0.1% gentamycin (Euroclone). All experiments were approved by the University of Campania “L. Vanvitelli” ethical committee. CD34^+^ cells were purchase from STEMCELL TECHNOLOGIES (US) Catalog # 70002.

### GFP pull-down assay and WB analysis

GFP pull-down assay was performed as previously described^32^.

For WB analysis, cells were lysed in RIPA buffer (1% Triton X-100, 0.1% SDS, 150 mM NaCl, 1 mM EDTA pH 8, 10 mM Tris-HCl pH 8, and 1% protease inhibitor cocktail (Roche, Italy) centrifuged for 15 min at 4 °C and heated for 5 min at 95 °C; 25 µg of protein extract was subjected to SDS-polyacrylamide gel electrophoresis, blotted on PVC membrane (Bio-Rad, USA) and incubated overnight with appropriate antibodies. Relative protein expression was detected by ECL Chemiluminescence method (Bio-Rad). Bands intensity were quantified by Image J analysis.

### qPCR and primers

RNA extraction was performed using TRIzol (Thermo Fisher Scientific) according to supplier’s instructions. Five hundred nanograms of RNA was reverse transcribed using SuperScript VILO DNA Synthesis Kit (Thermo Fisher Scientific), as described by the manufacturer’s protocol. Quantitative real-time PCR was carried out with a Bio-Rad iCycler iQ Real-Time PCR Detection System using iQ SYBR Green Supermix (Bio-Rad). Analysis was performed by ΔΔCt method.

Primers are listed in Table 2.

### Colony-formation assay

Colony-forming capability of leukemic U937 cells was assayed by MethoCult H4535 enriched without erythropoietin EPO (Stemcell Technologies, Canada), according to the manufacturer’s instructions. Cells (1 × 10^4^) were cultured in triplicate and number of colonies was scored after 2 weeks. Colonies quantification was performed as previously described^33^.

### AnnexinV–APC assay

AnnexinV–Allophycocyanin (APC) staining assay was purchased from BD Biosciences (USA) and used according to the manufacturer’s instruction.

### Antibodies, plasmids, and chemicals

Primary antibodies used for WB were as follows: anti-BRD9 (Bethyl Laboratories, A303-781A), anti-Cyclin E1 (Abcam, ab3927), anti-ERK1/2 (SantaCruz Biotechnology, SC-94), anti-GFP (Abcam, ab290), anti-p21 (Cell Signaling Technology, #2947), anti-PARP (Abcam, ab32138), anti-STAT5 (Abcam, ab209544), anti-pSTAT5 (Abcam, ab32364), anti-SOCS3 (Abcam, ab16030), anti-Actin (Abcam, ab3286), and anti-SMARCA4 (Abcam, ab4081). Primary antibody used for ChIP was anti-BRD9 (Active Motifs, 61538). Antibodies were used according to the manufacturer’s instructions.

Caspase8 Z-IETD and Caspase9 inhibitor Z-LEHD were purchased from R&D System and were used at 100 μM.

Lentiviral shRNAs targeting BRD9 (TRC0000131081, TRC0000127634) and scrambled control (TRC000035) plasmids were from Sigma MISSION human shRNA library (Sigma-Aldrich); pcDNA5_FRT plasmid was kindly provided by Professor M. Vermeulen; pcDNA5_FRT-BRD9 was generated by cloning the PCR-amplified coding sequence region of *BRD9* in KpnI-XhoI digested pcDNA5_FRT vector; psPAX2 and pMD2.G were kindly provided by Professor A. Baldini.

### Lentiviral production and cell transduction

HEK293FT cells were transfected using Lipofectamine 2000 reagent (Thermo Fisher Scientific) according to the manufacturer’s instructions.

For lentiviral production, HEK293FT cells were transiently co-transfected with lentiviral plasmid and lentiviral packaging plasmids psPAX2 and pMD2.G in a ratio of 3:2:3. Collected cell supernatant was centrifuged and the lentiviral particle pellet was resuspended in 200 µl DMEM.

For cell transduction, 50 µl DMEM containing lentiviral vectors was added to 1 × 10^6^ cells. Cells were selected in puromycin at a final concentration of 1 µg/ml for 7 days.

### Cell cycle and PI analysis

Cells (2.0 × 10^5^) were collected, washed with phosphate-buffered saline (PBS) once, and resuspended in hypotonic buffer (0.1% NP-40, 0.1% sodium citrate, 50 µg/µL PI, RNAse A). Cells were then incubated in the dark for at least 30 min and measured by FACS Calibur flow cytometer (Becton Dickinson). For PI analysis, cells were collected, washed with PBS twice, resuspended in PI buffer (0.2 µg/µL PI, PBS 1%) and measured on FACS Calibur flow cytometer. Flow cytometric data were analyzed using ModFit (Verity), FlowJo 9.3, and Cell Quest (Becton Dickinson) technologies.

### Generation of inducible HeLa cell line overexpressing BRD9

HeLa-FRT cell line constitutively expressing TetR was provided by Professor M. Vermeulen. Cells were transiently transfected by Lipofectamine 2000, according to the supplier’s instructions, with pcDNA5_FRT-BRD9 and pOG44 plasmids. GFP-BRD9 HeLa cells were then selected with 100 μg/mL hygromycin B (Thermo Fisher Scientific) and 3 μg/mL blasticidin S (Sigma-Aldrich). To induce GFP-BRD9 expression, cells were treated overnight with 1 μg/mL doxycycline (Sigma-Aldrich).

### Nucleofection of leukemic cells

Nucleofection of U937 and K562 cells was performed using Amaxa Nucleofector Kit C and Kit V (Lonza), respectively, according to the manufacturer’s protocol.

### Nuclear extraction and label-free GFP pulldown for MS analysis

Nuclear extraction was performed as previously reported^34^. Briefly, HeLa cells expressing GFP-BRD9 (GFP-BRD9 HeLa) and wild-type HeLa cells were colleced and homogenized using a tight pestle in the presence of 0.15% NP-40 (Roche) and complete protease inhibitors (Roche). Samples were then incubated in hypotonic buffer. The nuclei were pelleted by centrifugation and incubated with lysis buffer (420 mM NaCl, 0.1% NP-40, and complete protease inhibitors) for 1 h to extract nuclear proteins. The nuclear extract was obtained by a final centrifugation step at 13,000 r.p.m. for 30 min at 4 °C. The GFP-BRD9 HeLa and wild-type HeLa nuclear extracts were subjected to GFP pulldown using GFP-trap beads (Chromotek). For each pulldown, 1 mg of nuclear extract was incubated with 15 μL beads in incubation buffer (300 mM NaCl, 0.15% NP-40, 0.5 mM dithiothreitol, 20 mM HEPES–KOH pH 7.9) containing ethidium bromide at a final concentration of 50 mg/mL. Ethidium bromide was added to the reaction to prevent indirect, DNA-mediated interactions. Beads were then washed twice with incubation buffer containing 0.5% NP-40, twice with PBS containing 0.5% NP-40, and finally twice with PBS only.

### Sample preparation and MS analysis

Sample preparation and MS analysis were performed as previously described^35^. For MS data analysis, raw data were analyzed by MaxQuant software (version 1.5.1.0) using standard settings and the additional options match between runs, LFQ, and iBAQ. Volcano plots were generated as previously described^32^ using Perseus version 1.4.0.8 and in-house R scripts.

### RNA sequencing

RNA-seq and RNA-seq analysis were performed as previously reported^36^. The mass spectrometry proteomics data have been deposited to the ProteomeXchange Consortium via the PRIDE partner repository with the data set identifier PXD013245.

### Chromatin preparation

Chromatin preparation was essentially performed as previously described^37^ with some modifications. Briefly, U937 and HeLa cells were protein crosslinked using 2 mM Disuccinimidyl Glutarate (DSG) for 45 min at room temperature (RT). Cells were then washed twice with PBS and DNA crosslinking was performed with 1% formaldehyde for 15 min (U937) or 10 min (HeLa) at RT with gentle shaking. Crosslinking reaction was quenched using 1.25 M glycine. Cells were then washed with PBS twice and collected in Buffer B (20 mM HEPES, 0.25% Triton X-100, 10 mM EDTA, and 0.5 mM EGTA). Cells were pelleted by centrifuge at 2000 r.p.m. for 5 min at 4 °C and resuspended in Buffer C (150 mM NaCl, 50 mM HEPES, 1 mM EDTA, and 0.5 mM EGTA). After that, cells were pelleted and resuspended in 1× incubation buffer (0.15% SDS, 1% Triton X-100, 150 mM NaCl, 1 mM EDTA, 0.5 mM EGTA, and 20 mM HEPES) at 15 million cells/mL. Cells were sheared in a Bioruptor Pico sonicator (Diagenode) at 4 °C using 6 (U937) or 7 cycles (HeLa) of 30 s ON, 30 s OFF. Sonicated material was centrifuged at 13,000 r.p.m. for 15 min at 4 °C, then stored at −80 °C.

### Chromatin immunoprecipitation

Cells (10 × 10^6^) were used as input for library preparation and 5 × 10^6^ cells were used as input for ChIP-qPCR experiments. Chromatin was pre-cleaned by incubating with Protein A/G Dynabeads (Invitrogen) for 90 min at 4 °C in 1× incubation buffer (0.15% SDS, 1% Triton X-100, 150 mM NaCl, 1 mM EDTA, 0.5 mM EGTA, and 20 mM HEPES) supplemented with protease inhibitors and 0.1% bovine serum albumin (BSA). Chromatin was then incubated with 5 µg of antibody overnight at 4 °C in 1× incubation buffer supplemented with protease inhibitors and 0.1% BSA. Protein A/G Dynabeads were added the day after followed by a 90 min incubation. The beads were washed twice with Wash Buffer 1 (0.1% SDS, 0.1% sodium deoxycholate, 1% Triton, 150 mM NaCl, 1 mM EDTA, 0.5 mM EGTA, and 20 mM HEPES), once with Wash Buffer 2 (Wash Buffer 1 with 500 mM NaCl), once with Wash Buffer 3 (250 mM LiCl, 0.5% sodium deoxycholate, 0.5% NP-50, 1 mM EDTA, 0.5 mM EGTA, and 20 mM HEPES), and twice with Wash Buffer 4 (1 mM EDTA, 0.5 mM EGTA, and 20 mM HEPES). After washing, beads were rotated for 30 min at RT in Elution Buffer (1% SDS, 0.1 M NaHCO_3_). The supernatant was decross-linked with 200 mM NaCl and 100 μg/mL Proteinase K overnight at 65 °C. Decross-linked DNA was purified by MinElute PCR Purification columns (Qiagen). DNA amount was quantified using Qubit fluorometric quantitation (Thermo Fisher Scientific).

### ChIP-seq and RNA-seq processing

ChIP-seq reads were aligned using BWA-ALN mapper against the hg19 reference human genome. The reads marked as PCR duplicates and with mapping quality score <15 were removed from further analysis. Peak calling was performed by MASC2 using a *Q*-value cutoff of 0.01. Peaks were annotated using Homer software. ChIP-seq and RNA-seq data have been deposited in NCBI's Gene Expression Omnibus database and are accessible through GEO Series accession number: GSE129437.

RNA-seq reads were aligned using Bowtie mapper against the GRCh37.72 reference human transcriptome. The reads with mapping quality score <15 were removed from further analysis. Mapped transcripts were quantified using MMSEQ pipeline.

Downstream analyses were performed using R built-in packages, gplots, ggplot2, and fluff. GSEA analysis was performed against MSig cancer hallmark database.

Public ChIP-seq datasets used for this study are listed in the table below (Table 1).

**Table 1:** Public ChIP-seq datasets

ChIP	Cell line	GEO accession number
H3K4me3	U937	GSM1486000, GSM1486001
	HeLa, HeLa-Kyoto	GSM501713, GSM566169
H3K9K14ac	U937	GSM585614
	HeLa-Kyoto	GSM566174
POLD2A	U937	GSM726988
	HeLa	GSM1088665

### Primers

Primers used for this study are listed in the table below (Table 2).

**Table 2:** List of oligonucleotides used as PCR primers

Target	Sequences
*BRD9*	F5′-ATGTTCCATGAAGCCTCCAG-3′ / R5′-AGCTCCTTCTTCACCTTCCC-3′
*CDKN1A*	F5′-TTTCTACCACTCCAAACGCC-3′/ R5′-CGGCCAGGGTATGTACATGAG-3′
*MYB*	F5′-AAGCTACTGCCTGGACGAAC-3′ / R5′-CAGGGAGTTGAGCTGTAGGC-3′
*MKI67*	F5′-AGGCAAAGAAGACCTGCT-3′ / R5′-GAGAGTTTGCGTGGCCTGTA-3′
*PCNA*	F5′-CTGAGGGCTTCGACACCT-3′ / R5′-GTATCCGCGTTATCTTCG-3′
*HPRT*	F5′-TGAGGATTTGGAAAGGGT-3′ / R5′-CCTCCCATCTCCTCCATC-3′
*ACTIN*	F5′-CTCCTGAGCGCAAGTACT-3′ / R5′-CGTCATACTCCTGCTTGC-3′
*MYC*	F5′-ATTCTCTGCTCTCCTCGACG-3′ / R5′-CTGTGAGGAGGTTTGCTGTG-3′
*IRF9*	F5′-AGGAGGAAGAGGATGCCATG-3′ / R5′-TGCTGCTCCCAATGTCTGAA-3′
*DDIT3*	F5′-CTCTGGGAGGTGCTTGTGA-3′ / R5′-AACACTCTTGACCCTGCTTC-3′
*SOCS3*	F5′-AGACTTCGATTCGGGACCAG-3′ / R5′-GGAAACTTGCTGTGGGTGAC-3′
*BCL2*	F5′-ACAACATCACAGAGGAAGTAGAC-3′ / R5′-CAATCACGCGGAACACTTGA-3′
*TCF7L2*	F5′-GCAAAGGTCGTAGCTGAGTG-3′ / R5′-TTCGCTTGCTCTTCTCTGGA-3′
*TNFSRF14*	F5′-TGTAGTCAAGGTGATCGTCTC-3′ / R5′-GCAGGGCCTCAATGACTGT-3′
*STAT1*	F5′-GCATGAAATCAAGAGCCTGG-3′ / F5′-GTCTCGTGTTCTCTGTTCTG-3′
*IER3*	F5′-GAACCGAACCCAGCCAAAAG-3′ / R5′-ACACCCTCTTCAGCCATCAG-3′
*SOCS3 promoter* (chr17:76,355,927-76,356,482)	F5′-GCACACACCTGTAATCCCAC-3′ / R5′-ATGGAGTCTTGCTGTGTTGC-3′
*IRF9 promoter* (chr11:615,758-616,229)	F5′-GCACACACCTGTAATCCCAC-3′ / R5′-ATGGAGTCTTGCTGTGTTGC-3′
*CDKN1A enhancer* (chr6:36,648,195-36,648,503)	F5′-ACCTTGTAAGCCTCAGTCTCC-3′ / R5′-ACGTACACTGCATCACCTCA-3′
*IER3 promoter* (chr17:40,428,089-40,428,716)	F5′-GTTTCACAGTCCCCATGCAG-3′ /R5′-TCTGTGGAGGGGACAAATCA-3′
*GRM promoter* (chr7:85745583 + 85745689)	F5′-CCAGTGCCTTCTTCAATACCATTA-3′ / R5′-TCTTCTACTGTCTGAGAGTTGCCTAAA-3′
*ZWIT promoter* (chr10:58688620 + 58688733)	F5′-AGAACTGGAACCATCCTGTAGAGA-3′ / R5′-CTTGCCTTGGAGTTATTTTCCTAAC-3′
*SLITRK1 promoter* (chr13:83855843 + 83855948)	F5′-GTACGTGGTGTATTCTTCATGTGTG-3′ / R5′-TTGGCTGGGATAACACTTCTATGA-3′

### Statistical analysis

Statistical analysis was performed by two-tailed unpaired *t*-test. *P* < 0.05 was considered significant. All experiments were performed in three biological and three technical replicates (unless differently specified in the text).

## Supplementary information

Supplementary informations
