# Supplementary material for: BRD9 binds cell type-specific chromatin regions regulating leukemic cell survival via STAT5 inhibition
Source: Cell Death Dis. 2019 Apr 18;10(5):338. doi: 10.1038/s41419-019-1570-9 (PMC6472371; doi:10.1038/s41419-019-1570-9)
Supplement: Supplementary file 1 — Supplementary informations [file 41419_2019_1570_MOESM1_ESM.pdf]

Supplementary figure 1

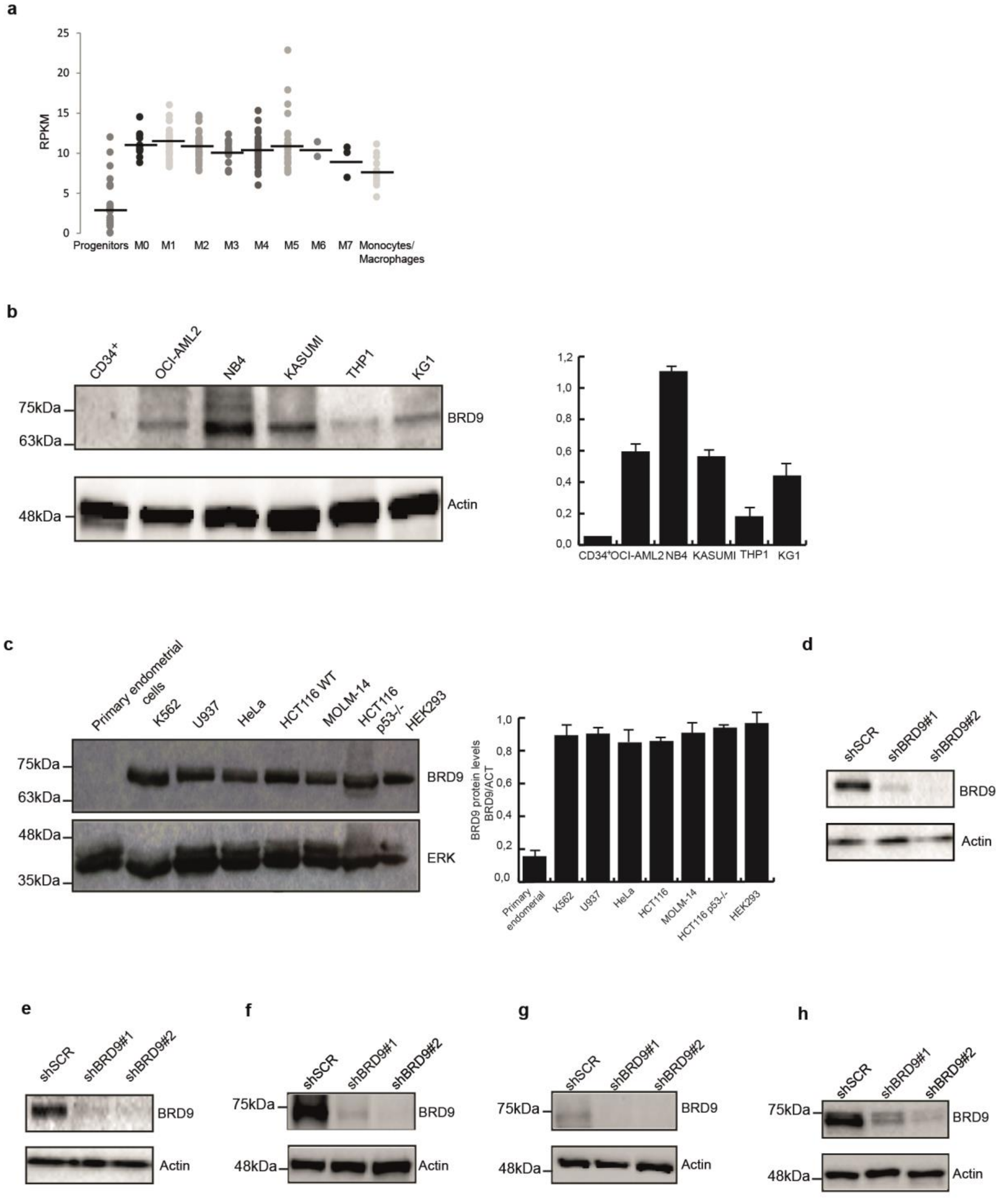

Supplementary Figure 1. **a** Dot plot bioinformatic analysis of BRD9 expression from publicly available RNA-seq data from 200 primary AML samples, 19 samples of human hematopoietic progenitors, and 16 samples of differentiated human monocyte and macrophage cells. Samples are

plotted based to WHO leukemic classification. β-Actin was used as housekeeping gene. **b** and **c** Western blot (WB) analysis showing BRD9 protein levels in different cancer cell (c) and a panel of AML cell lines (b) compared to non-tumorigenic cells (c) and $CD34^{+}$ respectively. ERK1/2 (c) and Actin (b) were used as loading control. Error bars indicate standard deviation (SD) of three biological replicates (*P <0.05, **P ≤0.01). **d**, **e, f**, **g** and **h** WB analysis showing depletion of BRD9 following shBRD9#1 and shBRD9#2 transduction in (d) NB4, (e) Kasumi (f) U937, (g) K562 and HL-60 (h) cells. Actin was used as loading control.

Supplementary figure 2

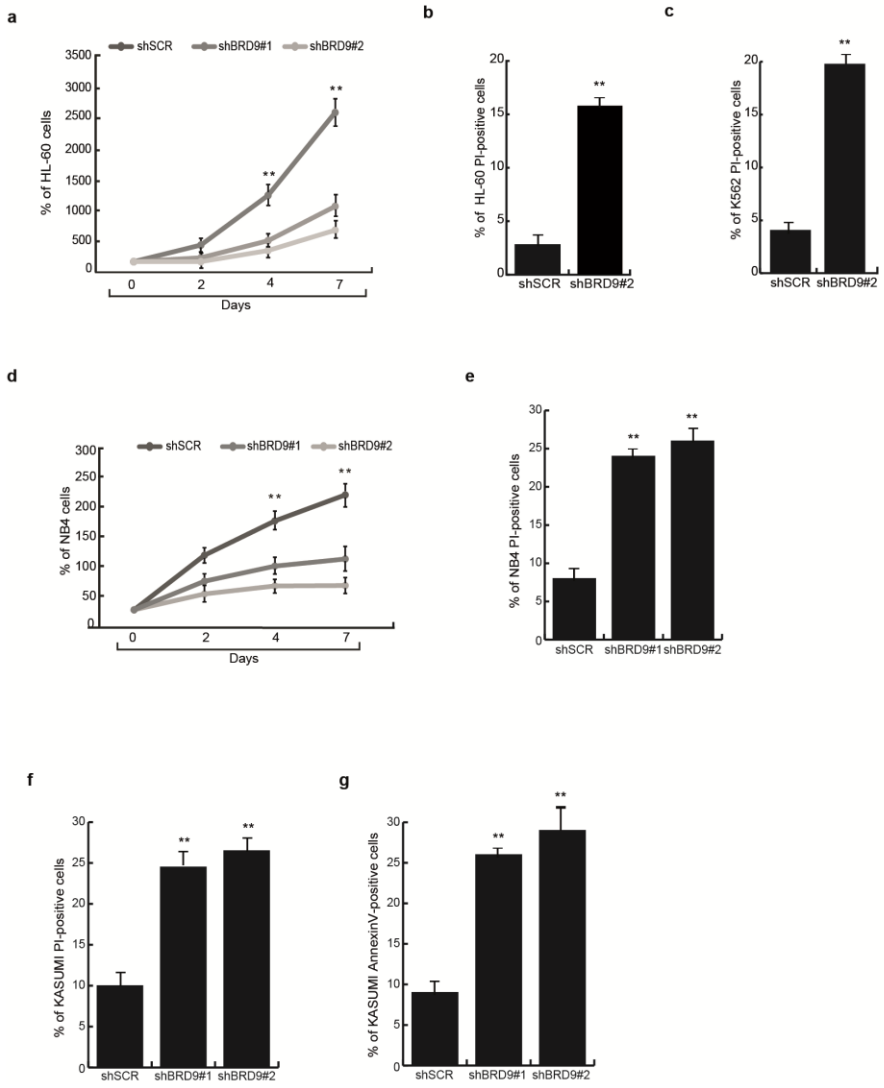

Supplementary Figure 2. **a** Competitive proliferation by trypan blue exclusion assay of shBRD9#1-, shBRD9#2- and shSCR-transduced HL-60 cells starting after 7 days of puromycin selection; error bars indicate SD of three biological replicates (**P* <0.05, ***P* ≤0.01). **b** and **c** Percentage of PI-positive cells following BRD9 depletion in HL-60 (b) and K562 (c) cells after 7 days upon puromycin selection (**P* <0.05, ***P* ≤0.01). **d** Competitive proliferation by trypan blue exclusion assay of shBRD9#1-, shBRD9#2- and shSCR-transduced NB4 cells after 5 days upon puromycin selection (**P* <0.05, ***P* ≤0.01). **e** and **f** Percentage of PI-positive cells following shBRD9#1-, shBRD9#2- and shSCR-transduced NB4 (e) and KASUMI (f) cells. Analysis was

performed after 5 days upon puromycin selection. error bars indicate SD of three biological replicates (**P* <0.05, ***P* ≤0.01). **g** Percentage of AnnexinV-positive KASUMI cells upon BRD9 knockdown; analysis was performed after 5 days upon puromycin selection; error bars indicate SD of three biological replicates (**P* <0.05, ***P* ≤0.01).

Supplementary figure 3

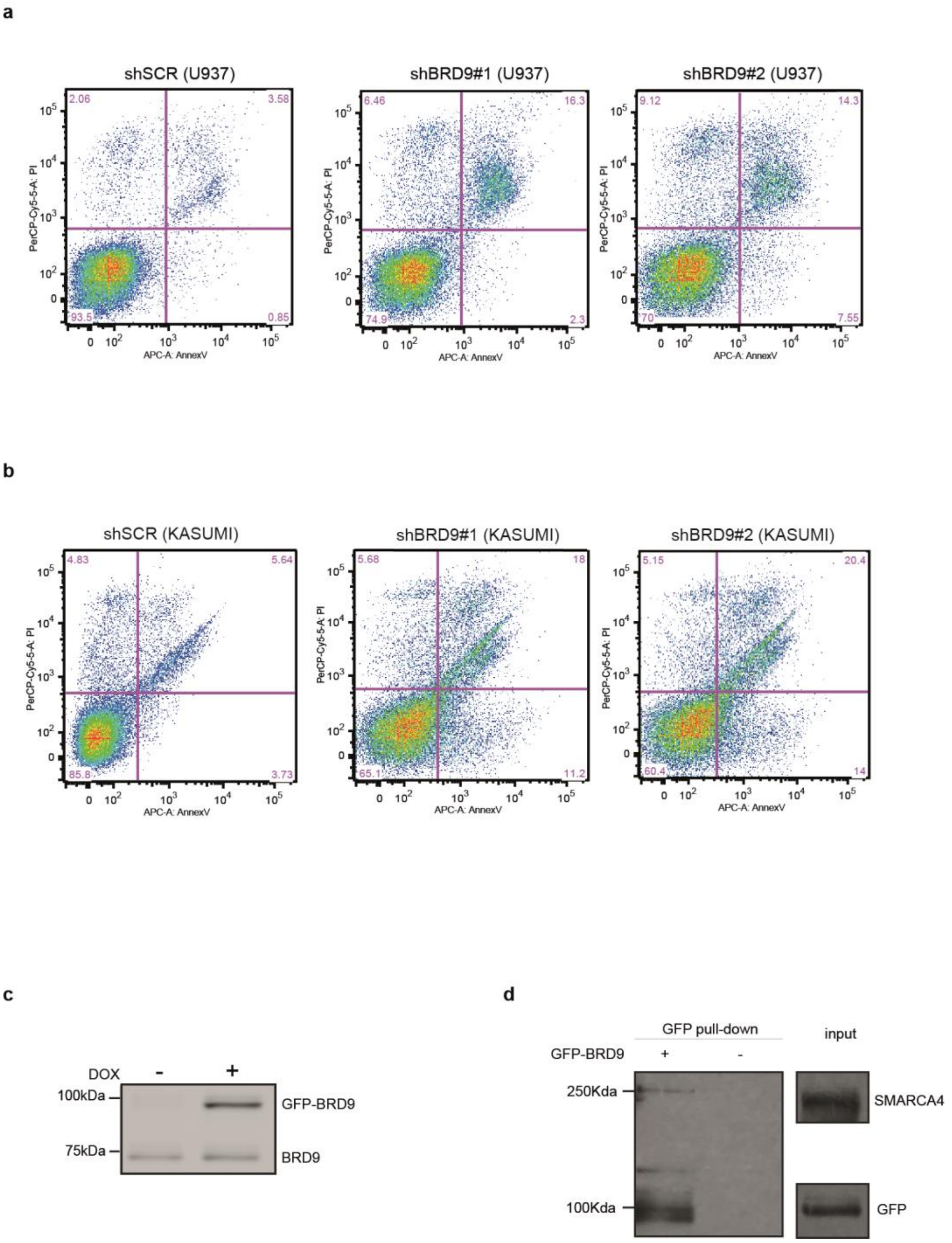

Supplementary Figure 3. a and **b** Representative AnnexinV experiment in U937 (a) and Kasumi (b) cell lines performed after 5 days upon puromycin selection. **c** WB analysis of indicated proteins following 16h upon doxycycline induction. **d** GFP pull-down of GFP-BRD9 following nucleofection of GFP-BRD9 expression plasmids in U937 cells. Immunoblotting was performed with the indicated antibody.

Supplementary figure 4

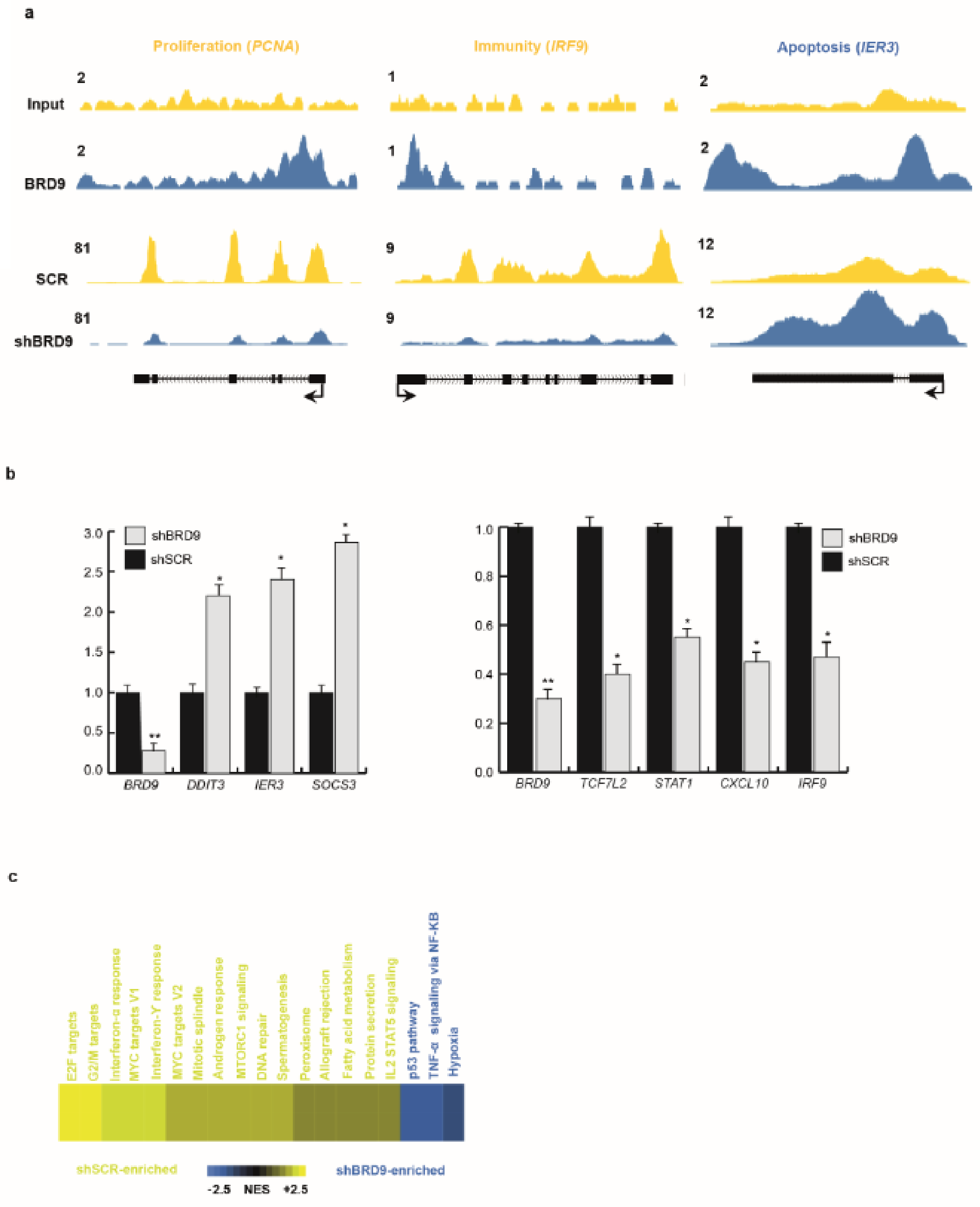

Supplementary Figure 4. **a** Representative differential genes with minimal 2-fold difference directly regulated by BRD9 promoter binding included in the top gene sets associated with BRD9 function. **b** RT-qPCR analysis of indicated genes following BRD9 depletion in U937 cells; error bars indicate SD of three biological replicates (**P* <0.05, ***P* ≤0.01). **c** GSEA analysis showing the top differential hallmark gene sets (nominal *P* <0.05) associated with SCR and shBRD9 cells (NES = normalized enrichment score).

Supplemetary figure 5

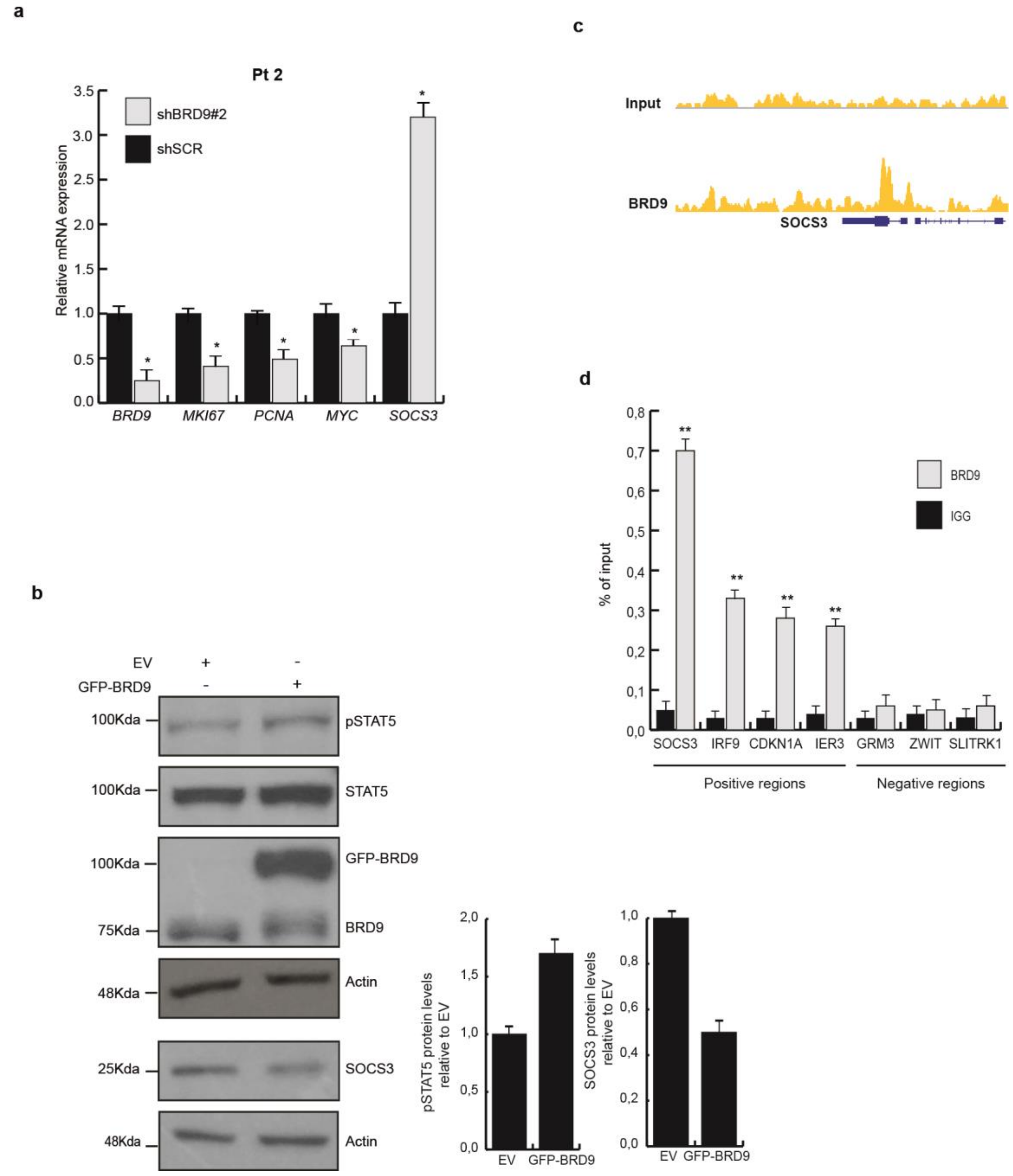

Supplementary Figure 5 a RT-qPCR showing relative expression levels of indicated genes. Experiment was performed 48 h following shBRD9 transduction of *ex vivo* leukemic cells (Pt 2). Error bars indicate SD of two biological replicates (**P* <0.05, ***P* ≤0.01). **b** WB analysis of indicated proteins 48 h following BRD9 overexpression in K562 cells. Actin was used as loading

control. Immunoblots were performed sequentially on the same membrane; error bars indicate SD of three biological replicates (**P* <0.05, ***P* ≤0.01). **c** BRD9 binding enrichment at SOCS3 promoter. **d** ChIP-qPCR experiment showing BRD9 enrichment at regulatory regions of indicated positive targets, no enrichment is observed for the negative targets. Results are express as percentage of input. Error bars indicate SD of three biological replicates (**P* <0.05, ***P* ≤0.01).
